# Cardiac troponin I levels and its relation to echocardiographic findings in infants of diabetic mothers

**DOI:** 10.1186/1824-7288-38-39

**Published:** 2012-09-04

**Authors:** Afaf Korraa, Mohamed Hesham Ezzat, Mahmoud Bastawy, Hassan Aly, Abdel-Azeem El-Mazary, Lobna Abd El-Aziz

**Affiliations:** 1 Pediatrics department, Faculty of Medicine for Girls, Al-Azhar University, Cairo, Egypt; 2 Cardiology department, faculty of medicine, Al-Azhar University, Cairo, Egypt; 3 Clinical-Pathology department, faculty of medicine, Al-Azhar University, Cairo, Egypt; 4 Pediatrics department, faculty of medicine, Minia University, Minia, Egypt

**Keywords:** Cardiac troponin I, Hypertrophic cardiomyopathy, Hypoglycemia, Infants of diabetic mothers, Interventricular septal dimension

## Abstract

**Background:**

Cardiomyopathy is noted in up to 40% of infants of diabetic mothers, and the exact mechanisms responsible for it are unknown. The aim of this study was to compare between infants of diabetic mothers (IDM) and infants of non- diabetic mothers (INDM) as regards cardiac troponin I (cTnI) levels (as a marker of cardiac dysfunction) and to examine the relationship between this marker and neonatal echocardiographic changes (cardiac structure and function).

**Methods:**

A prospective, comparative study included eighty full term neonates during the first three days of life, during the period from April 2008 to June 2011. They were divided into 2 groups, group I: included 40 infants of diabetic mothers (IDMs)and group II: included 40 infants of non diabetic healthy mothers as a control group.

**Results:**

37.5% of the IDMs were large for gestational age and demonstrated a significantly lower blood glucose level than the control group (34.6 ± 11.3mg/dl Vs 77.2 ± 19.8 mg/dl respectively) , respiratory distress and cyanosis were the presenting signs in 67.5% of IDMs. Cardiac TnI on the second day of life increased significantly in infants of diabetic mothers in comparison to INDM (p < 0.006) . IDMs had a significant increase in left atrial thickness ( 11.5 ± 1.8mm in IDM Vs 10.5 ± 0.9mm in INDM P < 0.002*) and a significant increase in septal thickness (5.0 ± 1.2mm in IDM Vs 4.0 ± 0.5mm in INDM P < 0.001*). CTnI correlated positively with interventricular septum thickness *(*P-value = 0.002*). Cardiac TnI was significantly increased in IDMs with respiratory distress (P *–*value < 0.05).

**Conclusions:**

This study demonstrated a highly significant positive correlation between cTnI level on the second day of life and the left ventricular end diastolic diameter (LVED) and interventricular septum diameter (IVSD). Cardiac troponin I (cTnI) is a useful biochemical marker for monitoring myocardial injury in infants of diabetic mothers. An elevated cTnI concentration in infants of diabetic mothers with respiratory distress was a good predictor for hypertrophic cardiomyopathy and/or left ventricular dysfunction.

## Background

Infant of a diabetic mother (IDM) is, defined as, a neonate born to mother who had suffering from diabetes mellitus, but this term refers specifically to the neonate born to a woman who had persistently elevated blood sugar during pregnancy [1]. Cardiovascular abnormalities in infants of diabetic mothers occur in the form of congenital heart diseases (3-5%), and cardiomyopathy (10-20%) [2]. It is suggested that fetal hyperinsulinism may trigger hyperplasia and hypertrophy of myocardial cells by increasing fat and protein synthesis [3,4].

Troponin is a calcium-regulated, three subunit proteins (Troponin C, Troponin I and Troponin T) involved in the actin-myosin interaction in muscle cells, inhibiting the ATPase activity of actomyocin, resulting in muscle relaxation [5]. The serum levels of cardiac troponin I (known to be a marker for cardiac injury) in infants of diabetic mothers, were significantly elevated in symptomatic patients with life threatening respiratory or homodynamic distress [6,7]. It is important to note that cardiac troponin I is a marker of all heart muscle damage, not just myocardial infarction. Troponins are increased in several forms of cardiomyopathy, such as hypertrophic cardiomyopathy or (left) ventricular hypertrophy, presumably due to increased oxygen demand and inadequate oxygen or blood supply to the heart muscle [8,9].

The aim of this study was to compare between infants of diabetic and those of non- diabetic mothers as regards cardiac troponin I (cTnI) levels (as a marker of cardiac dysfunction) and to examine the relationship between this marker and neonatal echocardiographic changes (cardiac structure and function).

## Methods

### Study design

This is a prospective, comparative study.

#### Population

Eighty full term neonates were included in this study. They were recruited from the neonatal intensive care units , AL-Zahraa and Al-Hussein university hospitals, during the period from April 2008 to June 2011. Sex, birth weight, mode of delivery, Apgar score, cord acid–base status, Downs Score (to assess the severity of respiratory distress) was recorded.

They were divided into 2 groups: group I included fourty infants of diabetic mother (IDMs), they were 25 males (62.5%) and 15 females (37.5%) and group II included 40 infants of non diabetic healthy mothers as a control group. They were 24 males (60%) and 16 females (40%).

Preterm neonates (less than 37 weeks), neonates with major central nervous system, cardiac and/or pulmonary anomalies, severe hypoxia, respiratory distress syndrome (RDS) or neonatal sepsis were excluded from this study. Mothers with history of hypertension, pre eclampsia, rheumatic heart disease or drugs except insulin for DM were excluded from the study. All deliveries were attended by a neonatologist expert in techniques of resuscitation. Apgar scoring at 1 and 5 minutes were recorded in all, and none of the neonates had birth asphyxia.

All studied groups were subjected to : full history taking, clinical examination, estimation of troponin I by using DRG troponin I fluorometric enzyme immunoassay (Centaur®, Bayer) [9]. cTnI was measured in the first and second days of life. The upper reference limits for cTnI, defined as the 95% cut-off by nonparametric analysis, were 0.1 ng/ml. cTnI values that exceed 1.5 ng/ml indicate myocardial necrosis compatible with acute myocardial injury.

#### Echocardiography

Neonatal echocardiography was performed during the first 3 days of life and cardiac measurements were determined by 2D and cross sectional M-mode echocardiography. Cardiac structure / function were assessed within the first three days of life by measuring left ventricle end-diastolic dimension (LVEDD), left ventricle end-systolic dimension (LVESD), ventricular septal thickness (VST), posterior wall thickness (PWT), percentage of ejection fraction (EF%) and percentage of shortening fraction (SF%).We used Hewlett Packard SONOS 5500 with a 5.0 or 3.5 MHZ transducer.

### Statistical analysis

Cardiac troponin I concentrations were not normally distributed and therefore medians and interquartile ranges are reported and non-parametric comparisons were made using the unpair comparison *t*-test (for normally distributed variables), Mann–Whitney *U* test (for non-normally distributed variables). Categorical variables were expressed in percentages. Categorical data were compared using a *χ*^2^ test. A Nonparametric Spearman ρ test was used to investigate the relationship between cTnI and cardiac function and structure parameters and Doppler indexes. P < 0.05 was considered statistically significant.

## Results

**Figure 1:**
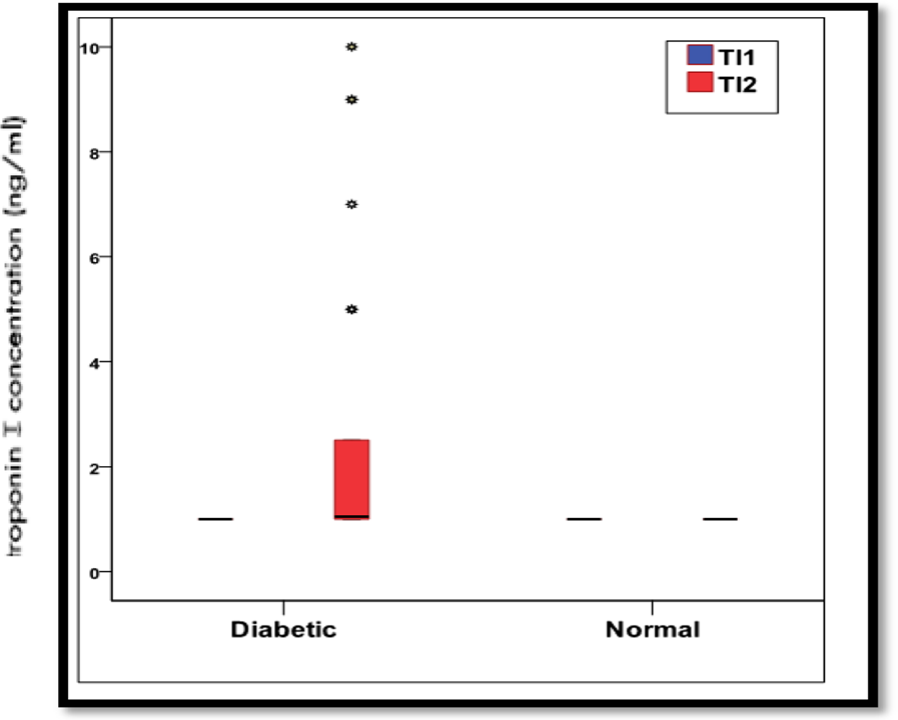
**Distribution of cardiac troponin I concentrations for infants of diabetic and non diabetic mothers.***The red box is the interquartile range, and the dark dots in the middle of the box is the median value*.

**Figure 2:**
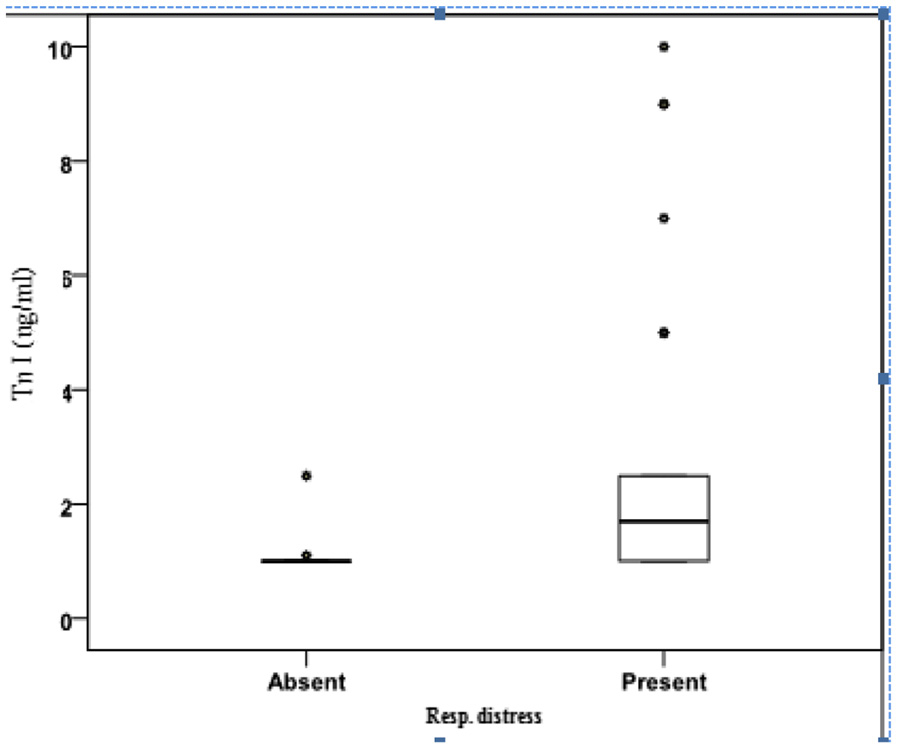
**Distribution of cardiac troponin I concentrations for infants of diabetic mothers with and without respiratory distress.***The box is the interquartile range, and the dark dots in the middle of the box is the median value*.

Among the diabetic mothers 32.5% were type-1 DM, 40% type-2 DM, and 27.5% were gestational diabetes. The demographic data of the studied groups are summarized in (Table 1). The mean weight of IDM was 4.1 ± 0.6Kg in comparison to 3.4 ± 0.2 Kg in INDM (P < 0.001*), the blood glucose level in IDMs showed a significantly lower level than INDM (34.6 ± 11.3mg/dl Vs 77.2 ± 19.8 mg/dl, P < 0.05* respectively). Respiratory distress and cyanosis were evident as a main clinical manifestations of IDMs in (67.5% and 60% respectively). On assessment of cTnI on the second day of life, it was significantly elevated in IDM (Table 2 & Figure 1), among them, infants with respiratory distress have a higher cTnI levels than those without (median values of 1.7ng/ml Vs 1.0 ng/ml respectively) (Table 3 & Figure 2).

**Table 1:** Comparison between diabetic and non-diabetic groups as regards to clinical data of neonates

	**IDM (Group1) No.40**	**INDM (Group 2) No.40**	**z**	**p**
**Gestational age (Weeks)**	36.7 ± 0.6	36.9 ± 0.9	1.169	0.245
**Birth wt** (Kg)	4.1 ± 0.6	3.4 ± 0.2	7.000	<0.001*
**APGAR 1**	5.8 ± 0.5	5.9 ± 0.3	1.085	0.281
**APGAR 2**	9.0 ± 0.0	9.0 ± 0.0	0.001	1.000
**Downs score** Median ( IQR)	1.0 (0.0-2.0)	0.5 (0.0-2.0)	-0.867^**&**^	0.386
**Sex**				
** Male**	25 (62.5%)	24 (60.0%)	0.00 *α*	1.00
** Female**	15 (37.5)	16 (40.0%)		
**Resuscitation**				
** Supportive**	15 (37.5%)	20(50.0%)	0.813 *α*	0.367
** Routine**	25 (62.5%)	20(50.0%)		
**RBS** (mg/dl)	34.6 ± 11.3	77.2 ± 19.8	11.818^#^	<0.001*

# Independent *t*-test *significant.

α Chi square test &Mann Whitney test.

*IDM*: Infant of diabetic mothers, *INDM*: Infant of non diabetic mothers.

*RBS*: Random Blood Sugar.

**Table 2:** cTnI in infants of diabetic and non diabetic mothers in the first and second days of life

	**INDMs Median (IQR)**	**IDMs Median (IQR)**	**P**	**z**
**TnI ng/ml (1**^**st**^**)**	1.0 (1.0-1.0)	1.0 (1.0-1.0)	0.000	1.000
**TnI ng/ml (2**^**nd**^**)**	1.2 (1.0-2.5)	1.0 (1.0-1.0)	-2.742	0.006*

1^st^ = (first day of life) 2^nd^ = (second day of life).

* = Significant *IQR* = Interquartile range.

*IDMs* = Infants of diabetic mothers, *INDMs* = Infants of non diabetic mothers.

**Table 3:** **Relation between TnI (2**^**nd**^**) and clinical manifestations in infants of diabetic mothers**

	**Present**		**Absent**		**z**	**P**
	**N**	**Median (IQR) ng/ml**	**N**	**Median (IQR) ng/ml**		
**Resp. distress**	27	1.7 (1.0-2.5)	13	1.0 (1.0-1.0)	−2.984	0.003*
**Cyanosis**	24	1.1 (1.0-2.5)	16	1.0 (1.0-1.8)	−1.685	0.092

*Significant *IQR* = Interquartile range.

**Figure 3:**
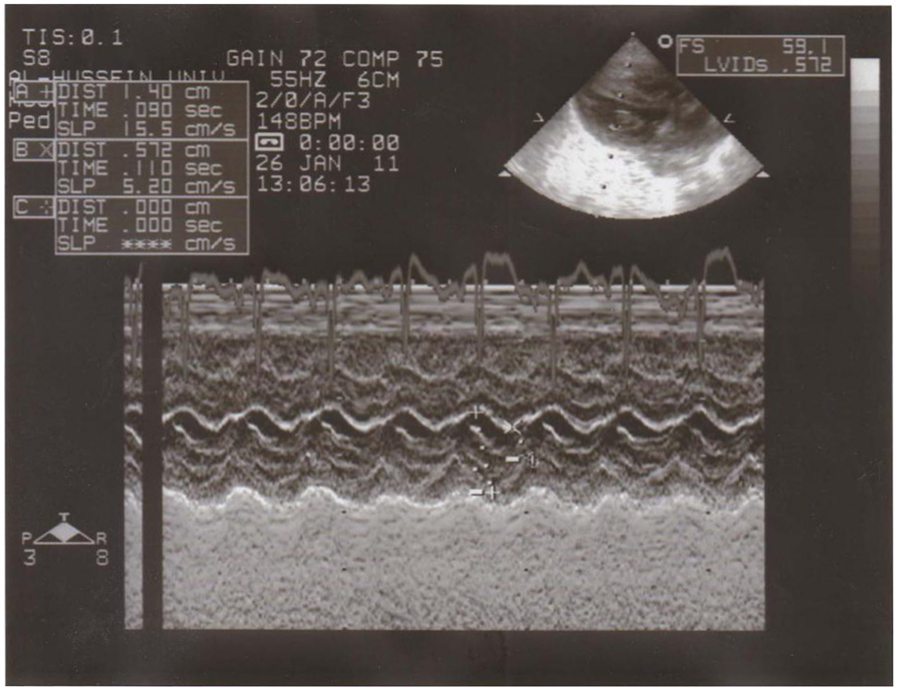
Correlation between cTnI and intraventriculer dimensions.

**Figure 4:**
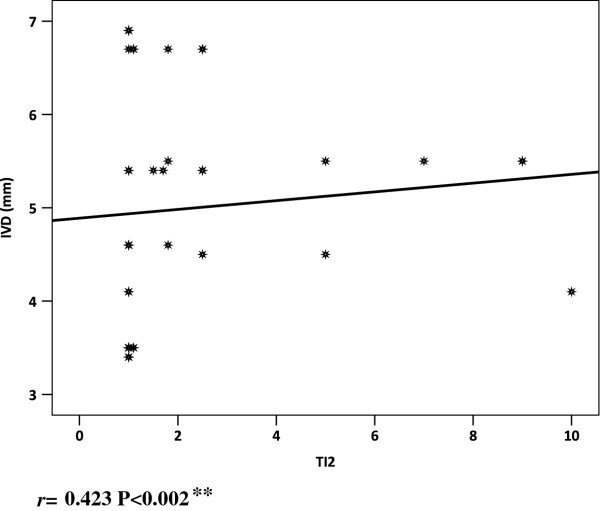
Correlation between cTnI and posterior wall thickness.

**Figure 5:**
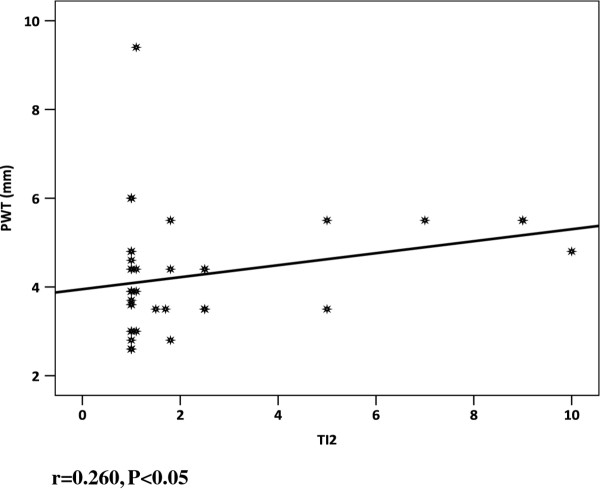
M-mode and parasternal long axis view for left atrial hypertrophy in an infant of a diabetic mother.

We found that the most common echocardiographic findings in IDMs were asymmetrical septal hypertrophy (80%), patent foramen ovale (PFO) (37.5%), and patent ductus arteriosus (PDA) (27.5%). We found a significant increase in left atrial thickness and interventricular septal dimension in infants of diabetic mothers in comparison to control (11.5 ± 1.8mm Vs 10.5 ± 0.9mm, and 5.0 ± 1.2mm Vs 4.0 ± 0.5mm respectively) (Table 4 & Figures 3,4,5). Cardiac TnI was significantly higher in IDMs with hypertrophic cardiomyopathy than without (median = 1.3 Vs 1.0 , P = 0.004** respectively) (Table 5).

**Table 4:** Echocardiographgic measurements of neonates in the studied groups

	**INDMs Mean ± SD**	**IDMs Mean ± SD**	**t ƛ**	**P**
**LVSD (mm)**	16.6 ± 2.1	16.6 ± 2.1	1.793	0.076
**LVDD (mm)**	10.5 ± 2.1	10.5 ± 2.1	1.601	0.113
**FS %**	37.4 ± 5.9	37.4 ± 5.9	0.816	0.416
**EF %**	70.5 ± 7.4	70.5 ± 7.4	2.928	0.0045
**PWT (mm)**	4.2 ± 1.3	4.2 ± 1.3	1.657	0.101
**LA (mm)**	11.5 ± 1.8	11.5 ± 1.8	3.143	0.0024*
**IVSD (mm)**	5.0 ± 1.2	5.0 ± 1.2	4.865	0.001*
**HCMP**	32 (80.0%)	32 (80.0%)	50.052	0.001*
**PFO**	15 (37.5%)	15 (37.5%)	0.813	0.367
**PDA**	11 (27.5%)	11 (27.5%)	2.625	0.105

* = Significant **ƛ** Independent *t*-test.

*IDMs* = Infants of diabetic mothers, *INDMs* = Infants of non diabetic mothers.

*IQR* = Interquartile range.

**Table 5:** Relation between cTnI and echocardiographic findings in neonates of diabetic mothers

	**Present**		**Absent**		**z∞**	**P**
	**N**	**Median (IQR)**	**N**	**Median (IQR)**		
**HCMP**	32	1.3 (1.0-2.5)	8	1.0 (1.0-1.0)	−2.897	0.004*
**PFO**	15	1.0 (1.0-1.0)	25	1.8 (1.0-3.8)	−3.127	0.002*
**PDA**	11	1.0 (1.0-2.5)	29	1.1 (1.0-2.5)	−1.006	0.315

* = Significant **∞** Mann Whitney test.

*IQR* = Interquartile range, *HCMP* = hypertrophic cardiomyopathy.

**Figure 6:**
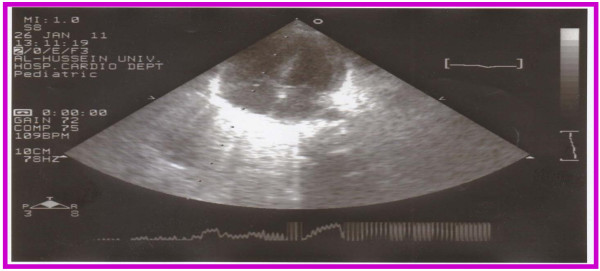
Apical four chamber view two-dimensional echocardiogram showing septal hypertrophy in an infant of diabetic mother.

**Figure 7:**
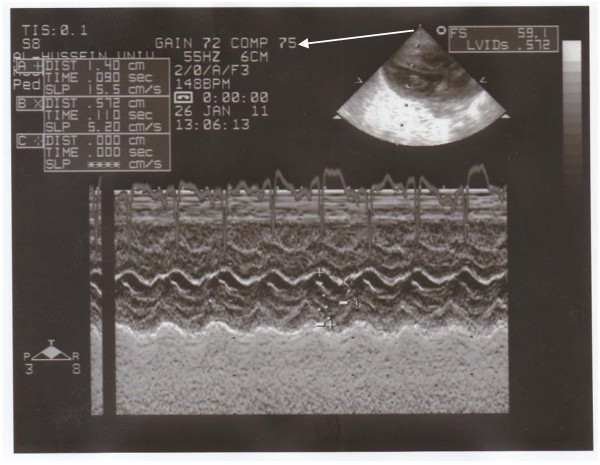
M-mode and parasternal long axis view showing interventricular septal hypertrophy in an infant of a diabetic mother.

There was a significant positive correlation between cardiac troponin I and both intraventriculer dimensions and posterior wall diameter in IDMs (*r =* ^0.423 P < 0.002^** & r = 0.260 P < 0.05 respectively) (Table 6, Figure 6,7). No significant correlations between cardiac troponin I and gestational age, birth weight, RBS, CRP, Apgar scores cord and acid–base status were recorded (Table 7). No relationship was observed between echocardiographic evidence of hypertrophic cardiomyopathy and maternal type of diabetes mellitus.

**Table 6:** **Correlation between TnI (2**^**nd**^**) and echocardiographic measurements of neonates**

	**r#**	**p**
**LVED (mm)**	0.306	0.031*
**LVES (mm)**	-0.020	0.891
**FS%**	0.143	0.322
**EF %**	0.177	0.218
**PWT (mm)**	0.260	0.050*
**LA D (mm)**	0.261	0.050*
**IVSD (mm)**	0.423	0.002*

* = Significant #Spearman correlation.

*LVED* : Left Ventrivular End Diastolic Dimension, *LVES*; Left Ventricular End Systolic Dimension.

*FS%* : shortening fraction, *EF%* : Ejection fraction, *PWT* : Posterior wall. Thickness, *LAD*: Left Atrium dimension, *IVSD* : Interventricular septal diameter.

**Table 7:** **Correlation between TnI (2**^**nd**^**) and both clinical & laboratory findings of infants of diabetic mothers**

	**r#**	**p**
Gestational age	0.106	0.463
Birth wt.	0.213	0.137
APGAR 1	−0.121	0.401
APGAR 2	0.000	1.000
RBS (mg/dl)	−0.214	0.136
CRP ( mg/L)	−0.112	0.439
PH (mmHg)	−0.035	0.812
PCO2 (mmHg)	−0.050	0.732
PO2 (mmHg)	0.174	0.227
HCo_3_ (mmol/L)	−0.165	0.253
BEe (mmol/L)	−0.156	0.280

*Significant #Spearman correlation.

*RBS*: Random Blood Suger, *CRP*: C- Reactive protein, *BE*: Base excess.

## Discussion

Diabetes is a relatively common condition in pregnancy, affecting up to 0.5% of the pregnant population [1]. Neonates of diabetic mothers are at increased risk of morbidity and mortality [2,3]. A well-recognized complication of diabetic pregnancy is the condition of hypertrophic cardiomyopathy. It was mentioned by vural et al. 1995 [5] that, while symptomatic hypertrophic cardiomyopathy occurs in 12.1% of infants of diabetic mothers, it is found in 40% when routinely searched for with an echocardiographic scan [5-7]. The primary aim of this study was to assess cardiac damage in infants of diabetic mothers, as assessed by cTnI levels and to determine whether there is a relationship between this cardiac marker and echocardiographic findings.

Troponin is an inhibitory protein complex found in all striated muscles, and the cardiac-specific isoform troponin I (cTnI) is a sensitive and specific marker of myocardial ischemia [10]. Our study showed that the cTnI concentration is unaffected by gestation, birth weight, sex, or mode of delivery, this is inconsistent with other publications [11]. In the current study, infants of diabetic mothers have increased plasma troponin I concentrations on the second day of life. Delivery and cardiovascular maladaptation to extra-uterine life can cause further compromise of oxygenation which lead to further myocardial compromise and troponin release [12]. Accordingly, elevations of troponin I was not detected immediately after birth.

Cardiac troponin I level was significantly higher in neoxnates who have respiratory distress and cyanosis compared to the controls. These findings may be explained in several ways. Firstly, myocardial injury with cardiac dysfunction may result in impaired gas exchange. Alternatively, respiratory and cardiac compromise may have a common cause as diabetes in the pregnant mother is strongly associated with neonatal cardiomyopathy [11-13]. Troponin release occurs in response to ischemia, and it is possible that diabetes-induced placental micro vascular disease and abnormal placentation may lead to chronic intrauterine hypoxia, which could affect fetal myocardial function, making infants of diabetic mothers more susceptible to an acute hypoxic insult [14-16].

The thickness of the interventricular septum was significantly more common in infants born to diabetic mothers. Hypertrophic cardiomyopathy was found in 80% of IDMs which is often transient with no lasting consequences for the majority of neonates [16-19]. In this study, an important number of infants presented with cardiorespiratory manifestations (67.5% with respiratory distress and 60 % with cyanosis). These factors reflect the problem of the higher incidence of cardiomyopathy in this study. Russell et al. 2008 [20] had reported previously that stillborn infants of diabetic mothers have heavier hearts than stillborn infants of non diabetic mothers after correction for fetal size, suggesting that cardiomyopathy may have a role in “unexplained” fetal death in diabetic pregnancy [19,20]. The etiology of this cardiomyopathy is poorly understood with proposed mechanisms including fetal hyperglycemia, hyperinsulinemia, and chronic hypoxia [20,21]. It has been suggested that cardiomyopathy occurs in response to functional changes evident in the first trimester in fetuses of pregestational type 1 diabetic mothers [22]. Early pregnancy hyperglycemia may also have an effect on the developing placenta and impaired placental functioning may result in chronic intrauterine hypoxia causing ischemic damage to the fetal myocardium and thus troponin release [23].

An increase in cTnI serum levels in infants with HCMP pointed to subclinical myocyte injury. The mechanisms of myocyte injury in HCMP are not fully understood. It may be caused by relative myocardial ischemia resulting from an imbalance between inappropriate hypertrophy of the myocardium and insufficient oxygen supply [20,22]. Left ventricular contractility as assessed by the ejection fraction and percentage shortening of the internal thickness was higher in infants of diabetic mothers compared with the controls. This finding could reflect myocardial compromise or an increase in the ventricular workload [24]. These data suggest that maternal diabetes is associated with significant effects on neonatal cardiac function with correlation between interventricular septal diameter and troponin I release in diabetic pregnancy. Poor glycemic control in early pregnancy changes fetal cardiac gene activation and predisposes the fetus to myocardial hypertrophy [24,25].

The findings in this work suggest that cTnI is a biochemical marker as sensitive as echocardiographic measurements in the detection of cardiac dysfunction and should be done to any infant born to a diabetic mother suffering from respiratory distress.

## Conclusions

Cardiac troponin I (cTnI) is a useful biochemical marker for monitoring myocardial injury in infants of diabetic mothers. It is a sensitive marker so as echocardiographic measurements in detection of cardiac dysfunction. It should be estimated in any infant born to a diabetic mother, mostly in those who present respiratory distress as it can predict hypertrophic cardiomyopathy and/or left ventricular dysfunction.

## Competing interests

The authors declare that they have no competing interests.

## Authors' contributions

All authors read and approved the final manuscript. Korraa A and El-Mazary conceived and designed the study and revised the manuscript for important intellectual content. They will act as guarantor of the study. M Ezzat, was responsible for performing echocardiography examination of children. M Bastawy and H Aly analyzed the data and helped in manuscript writing, revision and submission. Korraa A and El-Mazary helped in manuscript writing, revision and submission. Lobna conducted the laboratory tests and interpreted them.

## References

[B1] Aberg A Rydhstroem H Frid A Impaired glucose tolerance associated with adverse pregnancy outcome: a population-based study in southern Sweden Am J Obstet Gynecol 2001 184 77 83 10.1067/mob.2001.108085 11174484

[B2] McAuliffe FM Foley M Firth R Drury I Stronge JM Outcome of diabetic pregnancy with spontaneous labour after 38 weeks Ir J Med Sci 1999 168 160 163 10.1007/BF02945844 10540779

[B3] Evers IM de Valk HW Visser GH Risk of complications of pregnancy in women with type 1 diabetes: nationwide prospective study in the Netherlands BMJ 2004 328 915 920 10.1136/bmj.38043.583160.EE 15066886 PMC390158

[B4] Halliday H Hypertrophic cardiomyopathy in infants of poorly-controlled diabetic mothers Arch Dis Child 1981 56 258 263 10.1136/adc.56.4.258 7195689 PMC1627227

[B5] Vural M Leke L Mahomedaly H Maingourd Y Kremp O Risbourg B Should an echocardiographic scan be done routinely for infants of diabetic mothers? Turk J Pediatr 1995 37 4 351 356 8560603

[B6] Clark SJ Newland P Yoxall CW Subhedar NV Concentrations of cardiac troponin T in neonates with and without respiratory distress Arch Dis Child Fetal Neonatal Ed 2004 89 F348 F352 10.1136/adc.2002.025478 15210673 PMC1721706

[B7] Trevisanuto D Zaninotto M Altinier S Plebani M Zanardo V High serum cardiac troponin T concentrations in preterm infants with respiratory distress syndrome Acta Paediatr 2000 89 9 1134 1136 10.1080/713794580 11071098

[B8] Sato Y Taniguchi R Nagai K Makiyama T Okada H Yamada T Matsumori A Takatsu Y Measurements of cardiac troponin T in patients with hypertrophic cardiomyopathy Heart 2003 89 6 659 660 10.1136/heart.89.6.659 12748227 PMC1767695

[B9] Ebell MH White LL Weismantel D A systemic review of troponin T and I values as a prognostic tool for patients with chest pain J Fam Pract 2000 49 746 753 10947143

[B10] Ohman EM Armstrong PW Christenson RH Granger CB Katus HA Hamm CW O'Hanesian MA Wagner GS Kleiman NS Harrell FE Jr Califf RM Topol EJ Cardiac troponin T levels for risk stratification in acute myocardial ischemia. GUSTO IIA Investigators N Engl J Med 1996 335 18 1333 1341 10.1056/NEJM199610313351801 8857016

[B11] Shu-Chi M Wang L-J Chen Y-L Lin M-I Sung T-C Correlation of troponin I with perinatal and neonatal outcomes in neonates with respiratory distress Pediatr Int 2009 51 4 548 551 10.1111/j.1442-200X.2009.02817.x 19438830

[B12] Szymankiewicz M Matuszczak-Wleklak M Hodgman JE Gadzinowski J Usefulness of cardiac troponin T and echocardiography in the diagnosis of hypoxic myocardial injury of full-term neonates Biol Neonate 2005 88 19 10.1159/000084067 15731551

[B13] Clark SJ Newland P Yoxall CW Subhedar NV Sequential cardiac troponin T following delivery and its relationship with myocardial performance in neonates with respiratory distress syndrome Eur J. Pediatr. Feb 2006 165 2 87 93 10.1007/s00431-005-0001-3 16228245

[B14] Carlson RJ Navone A McConnell JP Burritt M Castle MC Grill D Jaffe AS Effect of myocardial ischemia on cardiac troponin I and T Am J Cardiol 2002 89 2 224 226 10.1016/S0002-9149(01)02206-8 11792348

[B15] McAuliffe F Mears K Fleming S Grimes H Morrison JJ Fetal cardiac troponin I in relation to intrapartum events and umbilical artery pH Am J Perinato 2004 l 21 147 152 10.1055/s-2004-823775 15085497

[B16] Oran B Cam L Başpinar O Baysal T Reisli I Peru H Karaaslan S Koç H Gürbilek M Cardiac troponin-I in the serum of infants of diabetic mothers Cardiol Young 2003 13 3 248 252 12903871

[B17] Jensen DM Damm P Moelsted-Pedersen L Ovesen P Westergaard JG Moeller M Beck-Nielsen H Outcomes in type 1 diabetic pregnancies Diabetes Care 2004 27 2819 2823 10.2337/diacare.27.12.2819 15562191

[B18] Russell NE Foley M Kinsley BT Firth RG Coffey M McAuliffe FM Effect of pregestational diabetes mellitus on fetal cardiac function and structure Am J Obstet Gynecol 2008 199 312 e311 e317 10.1016/j.ajog.2008.07.016 18771996

[B19] Prefumo F Celentano C Presti F De Biasio P Luigi Venturini P Acute presentation of fetal hypertrophic cardiomyopathy in a Type 1 diabetic pregnancy Diabetes Care 2005 28 8 2084 2087 16043770 10.2337/diacare.28.8.2084

[B20] Russell NE Holloway P Quinn S Foley M Kelehan P McAuliffe FM Cardiomyopathy and cardiomegaly in stillborn infants of diabetic mothers Pediatr Dev Pathol 2008 11 10 14 10.2350/07-05-0277.1 18237240

[B21] Sardesai MG Gray AA McGrath MM Ford SE Fatal hypertrophic cardiomyopathy in the fetus of a woman with diabetes Obstet Gynecol 2001 98 925 927 10.1016/S0029-7844(01)01455-7 11704206

[B22] Mehta A Hussain K Transient hyperinsulinism associated with macrosomia, hypertrophic obstructive cardiomyopathy, hepatomegaly and nephromegaly Arch Dis Child 2001 88 822 824 12937110 10.1136/adc.88.9.822 PMC1719631

[B23] Ostlund I Hanson U Bjorklund A Hjertberg R Eva N Nordlander E Swahn ML Maternal and fetal outcomes if gestational impaired glucose is not treated Diabetes Care 2003 7 2107 2111 12832321 10.2337/diacare.26.7.2107

[B24] Kozák-Bárány A Jokinen E Pentti K Tuominen J Rönnemaa T Ilkka V Impaired left ventricular diastolic function in newborn infants of mothers with pregestational or gestational diabetes with good glycemic control Early Hum Dev 2004 77 1–2 13 22 15113627 10.1016/j.earlhumdev.2003.11.006

[B25] Ora B Çam L Baspinar O Baysal T Reisli I Peru H Karaaslan S Koç H Gürbilek M Cardiac troponin-I in the serum of infants of diabetic mothers Cardiol Young 2003 13 248 254 12903871

